# AaABF3, an Abscisic Acid–Responsive Transcription Factor, Positively Regulates Artemisinin Biosynthesis in *Artemisia annua*

**DOI:** 10.3389/fpls.2018.01777

**Published:** 2018-11-28

**Authors:** Yijun Zhong, Ling Li, Xiaolong Hao, Xueqing Fu, Yanan Ma, Lihui Xie, Qian Shen, Sadaf Kayani, Qifang Pan, Xiaofen Sun, Kexuan Tang

**Affiliations:** Joint International Research Laboratory of Metabolic and Developmental Sciences, Key Laboratory of Urban Agriculture (South) Ministry of Agriculture, Plant Biotechnology Research Center, Fudan-SJTU-Nottingham Plant Biotechnology R&D Center, School of Agriculture and Biology, Shanghai Jiao Tong University, Shanghai, China

**Keywords:** *Artemisia annua* L., artemisinin biosynthesis, abscisic acid, ABA-responsive elements binding factor, transcription factor

## Abstract

Artemisinin is well known for its irreplaceable curative effect on the devastating parasitic disease, Malaria. This sesquiterpenoid is specifically produced in Chinese traditional herbal plant *Artemisia annua.* Earlier studies have shown that phytohormone abscisic acid (ABA) plays an important role in increasing the artemisinin content, but how ABA regulates artemisinin biosynthesis is still poorly understood. In this study, we identified that AaABF3 encoded an ABRE (ABA-responsive elements) binding factor. qRT-PCR analysis showed that *AaABF3* was induced by ABA and expressed much higher in trichomes where artemisinin is synthesized and accumulated. To further investigate the mechanism of AaABF3 regulating the artemisinin biosynthesis, we carried out dual-luciferase analysis, yeast one-hybrid assay and electrophoretic mobility shift assay. The results revealed that AaABF3 could directly bind to the promoter of *ALDH1* gene, which is a key gene in artemisinin biosynthesis, and activate the expression of *ALDH1*. Functional analysis revealed that overexpression of AaABF3 in *A. annua* enhanced the production of artemisinin, while RNA interference of AaABF3 resulted in decreased artemisinin content. Taken together, our results demonstrated that AaABF3 played an important role in ABA-regulated artemisinin biosynthesis through direct regulation of artemisinin biosynthesis gene, *ALDH1*.

## Introduction

Malaria is considered to be a great threat to public health in the world. According to World Health Organization (WHO), Malaria has caused 445000 deaths globally in 2016 (World Health Organization [WHO], 2017). This devastating parasitic disease is transmitted by protozoan parasites of the genus *Plasmodium* in the female *Anopheles* species mosquitoes (Cox, 2010). Researchers have found that artemisinin, extracted from a Chinese herbal plant *Artemisia annua*, displays a great effect on the combat of chloroquine-resistant *Plasmodium falciparum* (Carter and Mendis, 2002). The artemisinin-based combination therapy (ACT) has been proved to be the most effective treatment of multidrug-resistant *P. falciparum* malaria (World Health Organization [WHO], 2017), resulting in the fact that the demand on artemisinin production keeps increasing.

The main genes encoding the enzymes and the intermediate reaction steps in artemisinin biosynthesis have been studied. Generally, the artemisinin biosynthesis is taken place in two outer apical cells of multicellular glandular secretory trichomes (Duke and Paul, 1993; Olsson et al., 2009). The carbocation formation and cyclization of amorpha-4, 11-diene from farnesyl pyrophosphate (FPP), a linear isoprene precursor, initiate the whole biosynthesis process (Bouwmeester et al., 1999; Wen and Yu, 2011). This initial step is catalyzed by amorpha-4, 11-diene synthase (ADS). Then the amorpha-4, 11-diene is oxidized twice into artemisinic alcohol and artemisinic aldehyde, respectively by a cytochrome P450 enzyme, CYP71AV1 (Ro et al., 2006). Alcohol dehydrogenase (ADH1) participates in the oxidation of artemisinic alcohol into aldehyde as another specific catalyzer (Paddon et al., 2013). Artemisinic aldehyde is gradually converted to dihydroartemisinic aldehyde by artemisinic aldehyde Δ11 (13) reductase (DBR2) (Zhang et al., 2008). Dihydroartemisinic aldehyde is further oxidized to dihydroartemisinic acid (DHAA) by the catalysis of aldehyde dehydrogenase (ALDH1) (Teoh, 2009). Besides, CYP71AV1 and ALDH1 convert artemisinic aldehyde to artemisinic acid (Ro et al., 2006; Teoh, 2009). The last two non-enzymatic steps involve the formation of artemisinin from DHAA and artemisinic B from artemisinic acid respectively, both of which are proved to be light-introduced (Sy and Brown, 2002; Czechowski et al., 2016). Therefore, ADS, CYP71AV1, DBR2, and ALDH1 are considered to be key enzymes in artemisinin biosynthesis (Ikram and Simonsen, 2017).

Meanwhile, the low yield of artemisinin in *A. annua* (0.01–0.8%) severely limits the commercial usage of the drug (Abdin et al., 2003). In order to enhance the artemisinin content in the *A. annua*, an enormous amount of efforts have been made to study the regulation of artemisinin biosynthesis (Ikram and Simonsen, 2017). Several endogenous hormones such as methyl jasmonate (MeJA) and abscisic acid (ABA) have been found to be regulators of artemisinin biosynthesis by inducing expression of genes encoding transcription factors (TFs) in *A. annua*. For example, overexpression of *AaWRKY1*, *AaERF1/2*, *AaMYC2*, and *AaNAC1* can enhance the production of artemisinin mediated by MeJA (Yu et al., 2012; Jiang et al., 2016; Lv et al., 2016; Shen et al., 2016).

However, the mechanism of ABA regulating artemisinin biosynthesis is still unclear. ABA responsive genes are characterized by the existence of multiple ABA–responsive elements (ABREs; PyACGTG) in the promoters (Giraudat et al., 1994; Shen et al., 1996; Busk and Pages, 1998; Narusaka et al., 2003). ABRE-binding factors (ABFs), or ABA-responsive element binding proteins (AREB), play an important role in ABA signaling pathway, which belong to group-A subfamily of basic leucine zipper (bZIP) TFs (Choi et al., 2000; Uno et al., 2000). AabZIP1 phosphorylated has been reported by an ABA-responsive kinase AaAPK1. Overexpression of *AabZIP1* increases the content of artemisinin in transgenic plants (Zhang et al., 2015, 2017). Another ABA receptor AaPYL9 can also promote artemisinin biosynthesis (Zhang et al., 2013). Previous research has found that the expression of *ADS, CYP71AV1*, *DBR2*, and *ALDH1* can be stimulated by ABA (Zhang et al., 2015). ABA also increases artemisinin content in cell suspension culture (Zebarjadi et al., 2018).

In this study, *AaABF3* was identified from 86 bZIP TFs in *A. annua*. Based on hierarchical cluster analysis and homologous analysis, we deduced that *AaABF3* might regulate artemisinin biosynthesis, as a mediator of ABA signaling. Therefore, we cloned *AaABF3* and proved its positive role in artemisinin biosynthesis through function analysis. qRT-PCR and GUS assay of the promoter-GUS transgenic lines revealed that *AaABF3* expressed in trichomes, leaves, and stems. Subcellular localization of the AaABF3 protein showed its nucleus localization. Furthermore, we proved that AaABF3 directly bound to the G-box motif in *ALDH1’s* promoter by the dual-luciferase, yeast one-hybrid assay and EMSA, and thus activating the expression of *ALDH1* gene. Based on these analyses, we demonstrated that *AaABF3* mediated the ABA modulated artemisinin biosynthesis by direct regulation on *ALDH1* gene.

## Materials and Methods

### Plant Materials

*Artemisia annua* used in this research was “Huhao 1,” originated in Chongqing and further subjected to several years of selection in Shanghai. For *in vitro* culture, seeds of *A. annua* were treated as previously described (Shen et al., 2016). The seeds were cultured for 3 days at 4°C, and then transferred to the pots in a growth chamber with 16 h/8 h, light/dark photoperiod and 65% relative humidity at 24°C.

Seeds of *Nicotiana benthamiana* were sown directly on the soil mixture (vermiculite: perlite: peat moss 6:1:3), and the growth condition was the same as that of *A. annua*.

### ABA Treatment

Five-week-old plants of *A. annua* were sprayed with 100 μM ABA solution (Sigma-Aldrich), with 1aaa ethanol as the mock. Apical meristems of *A. annua* were gathered at 0, 1.5, 3, 6, 9 and 12 h after spraying. Each group had three leaf samples from independent plants as biological repeats. The samples were quickly frozen in liquid nitrogen and stored at -80°C for use.

### RNA Isolation and Reverse Transcription

Tissues (roots, stems, leaves, buds, flowers, and trichomes) of *A. annua* were collected from 5-month-old *A. annua* in the field. RNA extraction was carried out using an RNA prep pure Plant kit (Tiangen Biotech, China) following the manufacturer’s instructions. The reverse transcription of cDNA was performed using a PrimeScript RT Master Mix (Takara, Japan).

### Cloning and Analysis of *AaABF3*

The *AaABF3* gene was cloned after sequence alignment in the genome of *A. annua*. The open reading frame (ORF) of the *AaABF3* gene was amplified from AaABF3-F and AaABF3-R (Supplementary Table S1) using KOD Plus (Toyobo, Japan) and ligated into PLB vectors (Tiangen Biotech, China) following the manufacturer’s instructions. The cDNA from *A. annua* young leaves was used as the template.

### Quantitative Real-Time PCR (qRT-PCR)

The expression of genes was analyzed by using the SuperReal PreMix Plus SYBR-Green (Tiangen Biotech, China) in a LightCycler 96 qRT-PCR Machine (Roche, Switzerland). The *β-actin* of *A. annua* was used as internal control and each sample was repeated three times. Primer sequences used in this study were listed in Supplementary Table S1.

### Transformation of *A. annua*

The full length cDNA of *AaABF3* sequence was cloned into PHB vector under double CaMV35S promoter to generate *pHB-CaMV35S::AaABF3-sYFP::NOS* with the sYFP fused to the C-terminal of *AaABF3*. For RNAi experiments, a 171 bp fragment of *AaABF3* was cloned into PHB vectors. The resulting constructs were introduced into *Agrobacterium tumefaciens* strain EHA105 and genetically transformed into *A. annua* for further analysis.

### GUS Assay

The promoter region of *AaABF3* gene was amplified using KODFX (Toyobo, Japan) and cloned into pCAMBIA1391Z vector carrying *GUS* gene between *Pst*I and *BamH*I sites. The construct was genetically transformed into *A. annua*. GUS assay was carried out as described before (Jefferson et al., 1987).

### Subcellular Localization of AaABF3

The ORF of *AaABF3* gene without the terminator codon TAG was inserted to the PHB-YFP expression vector under the CaMV35S promoter to form a PHB-AaNAC1–YFP fusion protein. The plasmid and P19 protein were then transformed into *A. tumefaciens* strain GV3101 for transient expression in the epidermal cells of *N. benthamiana*. *Agrobacterium* cells were cultured in MS liquid medium to an OD600nm of 0.6 and injected into leaves of 6-week-old *N. benthamiana* after incubation at room temperature for 3 h. The YFP signals were imaged 60 h later using a TCS SP5-II confocal laser microscopy (Leica Microsystems, Germany).

### Dual-Luciferase Assay

The promoter of *ALDH1* was cloned into the vector pGreen0800-LUC under the 35S promoter. The reporter construct *proALDH1:LUC* was mixed with the effector strain GV3101 harboring *35S:AaABF3* in 1:1 ratio and infiltrated into *N. benthamiana*. The effector construct *35S: YFP* was used as a control. The infiltrated leaves of *N. benthamiana* were detected after 2 days, using Dual-Luciferase^®^ Reporter Assay System (Promega, United States)

### Yeast One-Hybrid Assay

The fragment containing G-box (CACGTT) was amplified from the promoter of *ALDH1* and cloned into the placZ vector as the bait. The ORF of *AaABF3* was fused with the GAL4 activation domain (AD) in the pB42AD vector by primers with *EcoR*I and *Xho*I restriction enzymes (Supplementary Table S1) as the prey. The prey and bait constructs were both introduced into yeast strain EGY48 and cultured on synthetic dropout SD/-Trp/-Ura medium at 30°C. The yeast cells were collected 2 d later and cultured on the 5-bromo-4-chloro-3-indolyl-β-D-galactopyranoside (X-gal) medium in dark for 24 h. The empty placZ and pB42AD vectors were used as controls.

### Electrophoretic Mobility Shift Assay

To express and purify the pCold and pCold-AaABF3 protein, the plasmids were transformed into the *E. coli* strain Rosetta (CxBio, China). The method of prokaryotic expression and purification was described before (Zhang et al., 2017).

A 50 bp sequence with G-box in the promoter of *ALDH1* was designed as the probe (Supplementary Table S1) in this assay. The reaction was performed by using DIG Gel Shift Kit, 2nd Generation (Roche, Switzerland) according to the manufacturer’s instructions. The purified AaABF3 protein and the probes were performed on a 5% polyacrylamide gel. The gel was blotted on a nylon membrane and detected by ChemDoc^TM^ Touch Imaging System (BIO-RAD, America). The pCold protein which was the vector of AaABF3 was used as a control.

### High-Performance Liquid Chromatography

Leaves of 3-month-old A. *annua* were cut and dried in 50°C for 24 h. Then the dry leaves were ground into powder samples weighing 1 g. The samples were extracted by methanol and treated by ultrasonic twice. The supernatants were harvested by centrifugation and filtered through a 0.25 μm membrane. The Waters Alliance 2695 HPLC system (Milford, America) was used to analyze the contents of artemisinin. The method of detection was described in the previous study (Lu et al., 2013).

## Results

### Isolation and Characterization of *AaABF3*

**FIGURE 1:**
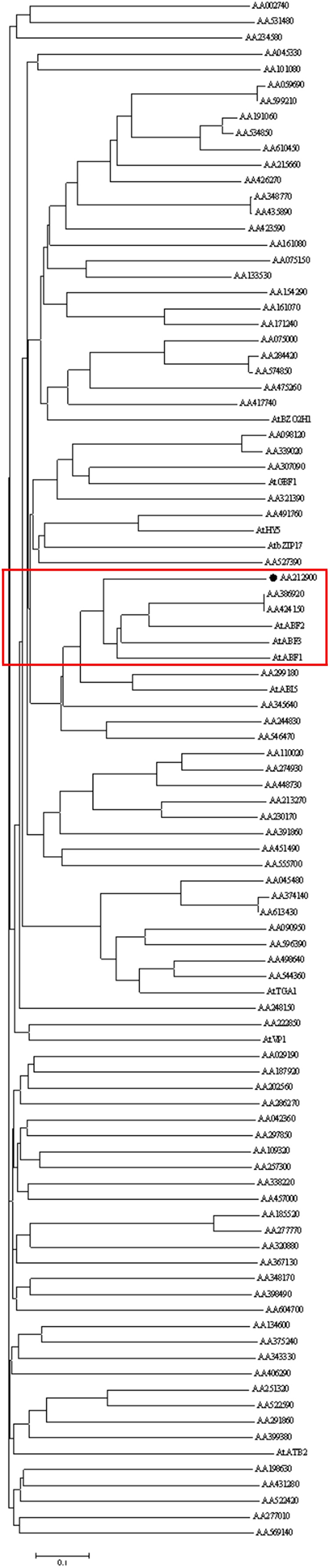
The phylogenetic tree of bZIP proteins in *Artemisia annua* and some typical bZIP proteins in *Arabidopsis thaliana*. The phylogenetic tree was constructed by using neighbor-joining method. The number of the bootstrap replicates 1000. The labeled protein AA212900 in red square was AaABF3. The sequences of AtBZO2H1, AtGBF1, AtHY5, AtbZIP17, AtABF2, AtABF3, AtABF1, AtABI5, AtTGA1, AtVP1, and AtATB2 were derived from NCBI (www.ncbi.nlm.nih.gov). Their corresponding GenBank accession numbers are as follows. AtABF1: NP_001185183.1, AtABF2: NP_001185157.1, AtABF3: NP_567949.1, AtABI5: NP_565840.1, AtATB2: NP_564761.1, AtbZIP17: NP_565946.1, AtBZO2H1: NP_849290.1, AtGBF1: NP_195391.1, AtHY5: NP_568246.1, AtTGA1: NP_201324.1, and AtVP1: NP_001320023.1.

In order to identify ABF genes that might be involved in artemisinin biosynthesis, we searched 86 bZIP TFs in *A. annua* by hierarchical cluster analysis. Previous study has reported that 10 bZIP TFs were clustered and had similar expression profiles with artemisinin biosynthetic pathway genes *ADS*, *CYP71AV1*, *DBR2* and *ALDH1* (Shen et al., 2018). A phylogenetic tree was built between 86 bZIP proteins and some typical bZIP proteins in *Arabidopsis thaliana* by using BioEdit (Figure 1). Only one gene showed high homology with ABFs, which implied that it might participate in artemisinin biosynthesis. Therefore, the full-length cDNA of this gene was isolated and named as *AaABF3.* The *AaABF3* gene contains an opening reading frame (ORF) of 1149 bp and encodes a protein of 382 amino acids. The theoretical isoelectric point of AaABF3 protein is 5.11 and the molecular weight is 92.18 kDa. AaABF3 shares a highly conserved bZIP superfamily domain, which can bind to DNA at the C-terminal region with other bZIP proteins (Supplementary Figure S1). A neighbor-joining tree of AaABF3 and other bZIP family members in different plant species were constructed by using MEGA 5. The phylogenetic tree showed that AaABF3 had the closest evolutionary relationship to CcABI5 (Supplementary Figure S2).

### Expression Profile of *AaABF3*

**FIGURE 2:**
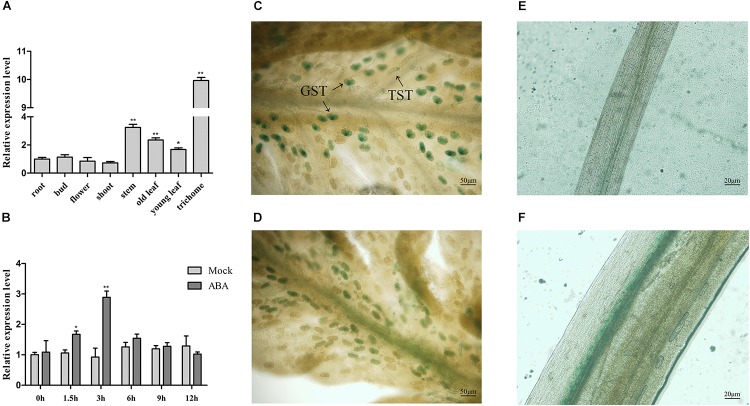
**(A)** Expression analysis of *AaABF3* in tissues of wild-type plants (WT). **(B)** Expression analysis of *AaABF3* in WT after ABA treatment. **(C–F)** GUS straining of 1-month-old *pAaABF3*-GUS transgenic *A. annua* plants in young leaf **(C)**, old leaf **(D)**, root **(E)**, and stem **(F)**. GST, glandular secretory trichome; TST, T-shaped trichome. The *β-actin* was used as control in **(A**,**B)**. Student’s *t*-test with *P* value determined whether the group difference was significant. ^∗^*P* < 0.05; ^∗∗^*P* < 0.01. Error bars represented the standard deviation (*n* = 3).

To detect whether *AaABF3* functions in artemisinin biosynthesis, the transcript level of *AaABF3* in different tissues was analyzed by qRT-PCR (Figure 2A). Results showed that *AaABF3* was highly expressed in trichomes, relatively high in leaves and stems, but low in roots, buds, flowers and shoots. The high expression of *AaABF3* in trichomes where the artemisinin was synthesized suggested that *AaABF3* might be involved in the artemisinin biosynthesis.

To further investigate the distribution of the AaABF3 protein, we constructed *pAaABF3*-GUS plasmid and transformed into wild type (WT). GUS straining of the transgenic plants revealed that the signal specifically concentrated in the trichomes (Figure 2C), leaf veins (Figure 2D) and stems (Figure 2E), but not in the roots (Figure 2F). *AaABF3* promoter drove the GUS expression mainly in the glandular secretory trichomes (GSTs) and non-glandular T-shaped trichomes (TSTs).

It is reported that the accumulation of artemisinin was stimulated by ABA (Jing et al., 2009). To investigate whether *AaABF3* was responsive to ABA, the expression of *AaABF3* after exogenous ABA treatment was analyzed by qRT-PCR. The results showed that the transcript of *AaABF3* increased rapidly after ABA treatment. The expression level reached the peak after 3 h, and declined to the original level at 9 h (Figure 2B), showing an obvious ABA-induced expression profile.

### Subcellular Localization of AaABF3

**FIGURE 3:**
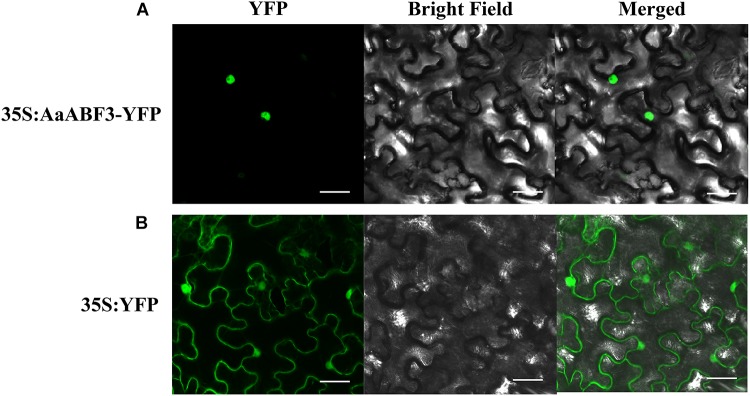
The subcellular localization of AaABF3 in leaves of *Nicotiana benthamiana*. **(A)** Localization of AaABF3 protein fused with YFP. **(B)** Localization of empty vector. Bars = 40 μm.

A yellow fluorescent protein (YFP) was fused to the N-terminus of AaABF3 to check the subcellular localization of AaABF3. The YFP fluorescence of 35S: AaABF3 -YFP was observed exceptionally in the nucleus (Figure 3A), while that of the control YFP was detected throughout the whole tobacco cell (Figure 3B). This indicated that AaABF3 was localized in the nucleus.

### Direct Regulation of AaABF3 on the *ALDH1* Gene

**FIGURE 4:**
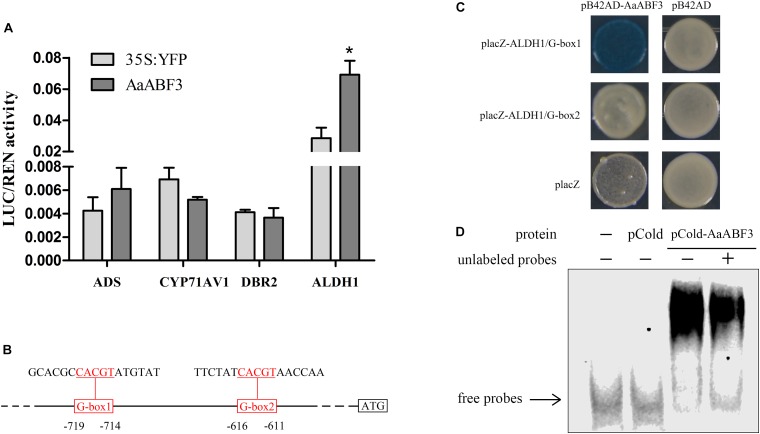
**(A)** Transient dual-LUC detected in tobacco leaves. The values were calculated by the ratio of firefly luciferase activities to renilla luciferase activities (LUC/REN). Student’s *t*-test with *P* value determined whether the group difference was significant. ^∗^*P* < 0.05. Error bars represented the standard deviation (*n* = 3). **(B)** Sketch map of the positions of two G-boxes in the *ALDH1* promoter which were triple repeated in the probes and used in yeast one-hybrid assay. The G-box motifs were marked red and underlined. **(C)** Yeast one-hybrid assay of AaABF3 and G-box motifs in promoter of *ALDH1*. Right columns represented the yeast cells grown on X-gal plates. PB42AD and placZ empty vector were used as negative controls. **(D)** EMSA assay of pCold-AaABF3 and G-box 1 motif of *ALDH1* promoter. The pCold-AaABF3 and pCold protein were expressed and purified. The labeled probes were derived from the G-box1 promoter region. Unlabeled probes were used as cold competitors (10X dilution compared with labeled probes). The empty pCold vector protein was used as a control.

Now that *AaABF3* influenced expression of artemisinin biosynthesis genes, and the nucleus-localized character of AaABF3 indicates that it might function as a transcription factor, we further detected the possible direct binding of AaABF3 to the promoter of *ALDH1*. The dual-LUC assay was carried out to investigate whether AaABF3 activated the transcription of key enzyme genes in artemisinin biosynthesis, such as *ADS, CYP71AV1, DBR2*, and *ALDH1*. Results showed that AaABF3 only improved the expression level of *ALDH1* promoter, up to 2.4 fold as compared with the 35S:YFP control (Figure 4A), indicating the activation of *ALDH1* by AaABF3 *in vivo*.

The activation of *ALDH1* promoter by AaABF3 indicated that there should be AaABF3 binding motifs, such as G-box (Choi et al., 2000), in the promoter region of *ALDH1*. Analysis of the *ALDH1* promoter revealed that there were two G-boxes (Figure 4B). We carried out yeast one-hybrid (Y1H) assay to check if AaABF3 could bind to these two motifs. The result showed that AaABF3 could only bind to the G-box1 motif in the *ALDH1* promoter (Figure 4C).

To further confirm the binding activity of AaABF3 and *ALDH1* promoter, an electrophoretic mobility shift assay (EMSA) was performed (Figure 4D). The shifted bands were detected with purified AaABF3 protein and G-box of ALDH1 promoter. The unlabeled probes were used as competitors to examine the DNA-binding specificity. These results showed that AaABF3 had a direct interaction with ALDH1 promoter at the G-box motif.

### Function of *AaABF3* in Artemisinin Biosynthesis

**FIGURE 5:**
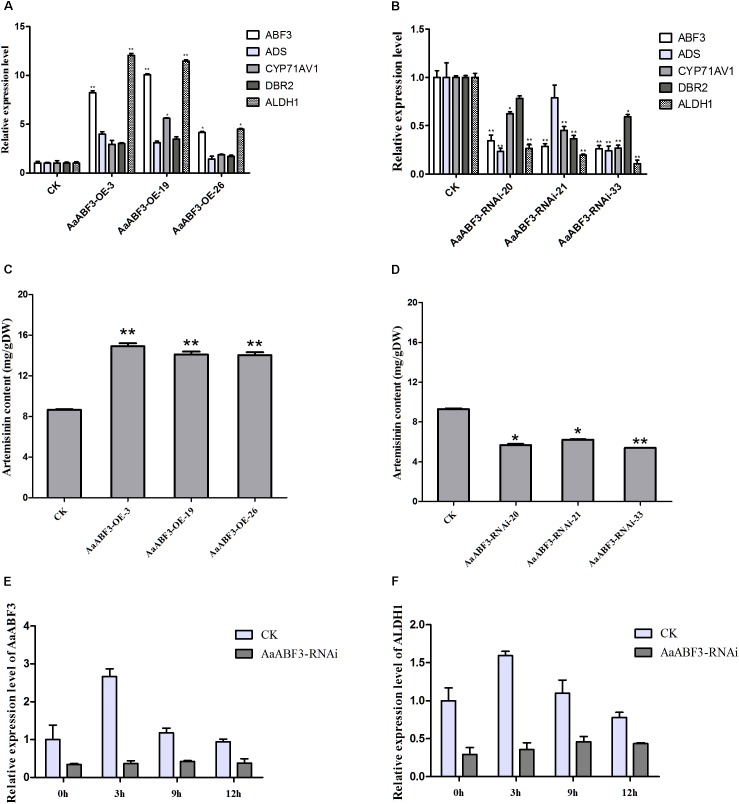
**(A**,**B)** Expression analysis of *AaABF3, ADS*, *CYP71AV1*, *DBR2*, and *ALDH1* in 3-month-old *AaABF3*-OE transgenic *A. annua* plants **(A)** and *AaABF3*-RNAi transgenic *A. annua* plants **(B),** respectively. **(C**,**D)** Contents of artemisinin in leaves of 3-month-old *AaABF3*-OE transgenic *A. annua* plants **(C)** and *AaABF3*-RNAi transgenic *A. annua* plants **(D),** respectively as determined by HPLC. **(E,F)** Expression analysis of *AaABF3* **(E)** and *ALDH1* **(F)** in *AaABF3*-RNAi transgenic *A. annua* plants at 0, 3, 9, and 12 h after ABA treatment. The *β-actin* was used as control in **(A**,**B**,**E**,**F)**. Student’s *t*-test with *P* value determined whether the group difference was significant. ^∗^*P* < 0.05; ^∗∗^*P* < 0.01. Error bars represented the standard deviation (*n* = 3).

Since AaABF3 activated *ALDH1* directly, we further analyzed whether *AaABF3* took a positive role in the artemisinin biosynthesis. We generated *AaABF3* overexpression transgenic *A. annua* plants, AaABF3-OE. As revealed by the qRT-PCR analysis, the transcription level of *AaABF3* was increased 4–10 fold in the transgenic plants (Figure 5A). It was also found that the transcription levels of key genes in artemisinin biosynthesis including *ADS, CYP71AV1, DBR2*, and *ALDH1* were increased by 1.4–3.3 fold, 1.9–5.6 fold, 1.7–3.5 fold and 5.0–12.0 fold, respectively in the AaABF3-OE plants. HPLC analysis showed that artemisinin contents in *AaABF3*-OE plants were 19–72% higher than that in the WT (Figure 5C).

Furthermore, in order to test whether the down-regulation of the expression of *AaABF3* though RNA interference (RNAi) could influence artemisinin biosynthesis, we generated the *AaABF3*-RNAi *A. annua* plants. It was found that the transcription levels of *AaABF3* in the *AaABF3*-RNAi lines were decreased to 20–34% compared with which in the WT, while those of *ADS, CYP71AV1, DBR2* and *ALDH1* were reduced to 23–78%, 27–62%, 37–78%, and 11–27%, respectively (Figure 5B). Accordingly, the artemisinin contents in the *AaABF3*-RNAi lines were also reduced to 58–78% compared with that in the WT (Figure 5D). In *AaABF3*-RNAi transgenic plants, the expression levels of *AaABF3* and *ALDH1* also increased, but to a lower extent than those in the WT (Figures 5E,F). These results demonstrate that *AaABF3* has a positive function on the artemisinin biosynthesis by up-regulating the transcription level of *ALDH1* and ABA promotes the biosynthesis of artemisinin throughout *AaABF3*.

## Discussion

ABF3 is an ABRE-binding protein, which is induced by environmental stress and requires ABA for its full activity (Choi et al., 2000; Uno et al., 2000). To further investigate the possible involvement of ABF3 protein in artemisinin biosynthesis, *AaABF3* was isolated after hierarchical cluster analysis and homology analysis. ABA treatment verified the sensitivity of *AaABF3* to ABA. GUS assay and tissues expression verified the trichome predominantly expression of *AaABF3*. Further function analysis and mechanism study revealed that *AaABF3* positively regulate artemisinin biosynthesis by directly regulating the functional gene *ALDH1* in artemisinin biosynthesis.

Although AaABF3 can only activate the promoter of *ALDH1*, the expression of *ADS*, *CYP71AV1*, and *DBR2* are also altered by the over-expression or down regulation of *AaABF3*. This indicates that AaABF3 should also regulate other artemisinin biosynthesis genes indirectly. It was reported that *ADS* and *CYP71AV1* was up-regulated by ABA via *AabZIP1* (Zhang et al., 2015). An ABA-responsive kinase, AaAPK1, is also involved in regulating artemisinin biosynthesis through phosphorylating itself and *AabZIP1* (Zhang et al., 2017). It is possible that *AaABF3* regulates other genes with the help of ABA-related genes except for AaAPK1. *ALDH1* catalyzes dihydroartemisinic aldehyde to DHAA, which is a requisite precursor for the generation of artemisinin. However, regulation of *ALDH1* gene is rarely reported. In this study, we demonstrate that AaABF3 binds to the promoter of *ALDH1* and activates its expression, thus promoting artemisinin biosynthesis. However, the expression level of *ALDH1* still increases in *AaABF3*-RNAi plants after ABA treatment, hence we suppose that there are other TFs also regulating *ALDH1* which we haven’t found yet. This is the first time that we report the mediation of *AaABF3* in ABA-regulated artemisinin activation and reveal the possible role of bZIP transcription factors in coordination of artemisinin biosynthesis.

Artemisinin is reported to be synthesized in yeast (Ro et al., 2006). However, *A. annua* is still the main source of artemisinin production in industry. Therefore, there is a great need to promote the production of artemisinin in *A. annua* to meet the growing demand for ACT treatment. The role of transcription factors in artemisinin promotion has been proved to be effective, and application of transcription factors has also been developed (Muangphrom et al., 2016). Previous study have shown that many transcription factors can promote artemisinin biosynthesis by activating the promoters of *ADS* and *CYP71AV1* (Yu et al., 2012; Zhang et al., 2015; Shen et al., 2016). This study demonstrates that the transcription factor *AaABF3* functions positively in the artemisinin promotion and can be applied to the engineering of artemisinin biosynthesis in the future.

## Accession Numbers

*AaABF3*: MH734935, AtABF1: NP_001185183.1, AtABF2: NP_001185157.1, AtABF3: NP_567949.1, AtABI5: NP_565840.1, AtATB2: NP_564761.1, AtbZIP17: NP_565946.1, AtBZO2H1: NP_849290.1, AtGBF1: NP_195391.1, AtHY5: NP_568246.1, AtTGA1: NP_201324.1, AtVP1: NP_001320023.1, CcABI5: XP_024960144.1, CsbZIP8: AGG39700.1, EgTRAB1: XP_010066019.1, HaABF4: XP_021993119.1, HaABI5: XP_021993117.1, LsABI5: XP_023754710.1, and PvbZIP6: AGV54705.1.

## Author Contributions

YZ and KT designed the research. YZ, LL, and XH performed the experiments. YZ, XH, XF, YM, and LX carried out expression analysis, vector construction, transgenic plant generation, subcellular localization, dual-luciferase, yeast one hybrid assay, and EMSA. YZ drafted the manuscript. QS, SK, QP, XS, and KT revised the manuscript. All authors approved the manuscript.

## Conflict of Interest Statement

The authors declare that the research was conducted in the absence of any commercial or financial relationships that could be construed as a potential conflict of interest.
