# Supplementary material for: AaABF3, an Abscisic Acid–Responsive Transcription Factor, Positively Regulates Artemisinin Biosynthesis in Artemisia annua
Source: Front Plant Sci. 2018 Nov 28;9:1777. doi: 10.3389/fpls.2018.01777 (PMC6279931; doi:10.3389/fpls.2018.01777)
Supplement: Supplementary file 1 [file Table_1.DOC]

***Supplementary Material***

**AaABF3, an abscisic acid‐responsive transcription factor, positively regulates artemisinin biosynthesis in *Artemisia annua***

**Yijun Zhong, Ling Li, Xiaolong Hao, Xueqing Fu, Yanan Ma, Lihui Xie, Qian Shen, Sadaf Kayani, Qifang Pan, Yueli Tang, Xiaofen Sun and** **Kexuan Tang***

***Correspondence:**Professor Kexuan Tang
[kxtang@sjtu.edu.cn](mailto:kxtang@sjtu.edu.cn)


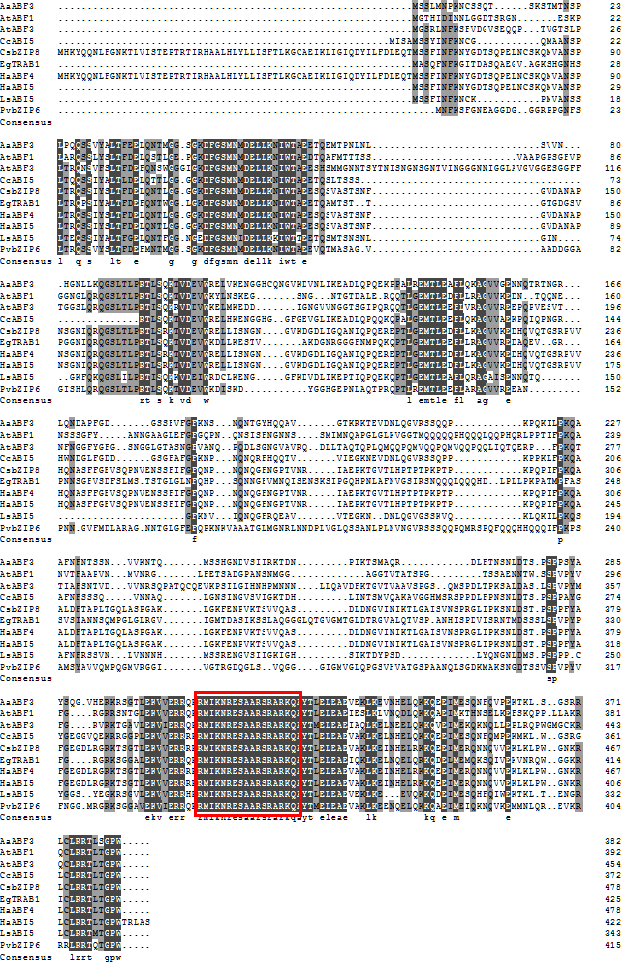


Fig. S1 Amino acid sequence alignment of AaABF3 and other bZIP proteins performed by DNAMAN. The selected bZIP proteins were the same as in Fig.1. The consensus sequence was showed in the last line. The amino acid residues shaded in black represented 100% homology, and the grey represented more than 75% homology. The sequence in red square was the bZIP DNA-binding and dimerization domain.

Fig. S2 The phylogenetic tree of AaABF3 and other bZIP proteins in *Arabidopsis thaliana*, *Camellia sinensis, Cynara cardunculus*, *Eucalyptus grandis, Helianthus annuus*, *Lactuca sativa* and *Phaseolus vulgaris*. The phylogenetic tree was constructed using neighbor-joining method. The number of the bootstrap replicates 1000. The bZIP proteins and their corresponding GenBank accession numbers are as follows. AtABF1: NP_001185183.1, AtABF2: NP_001185157.1, AtABF3: NP_567949.1, AtHY5: NP_568246.1, CcABI5: XP_024960144.1, CsbZIP8: AGG39700.1, EgTRAB1: XP_010066019.1, HaABF4: XP_021993119.1, HaABI5: XP_021993117.1, LsABI5: XP_023754710.1, and PvbZIP6: AGV54705.1.

Table S1 Primers used in the study

| Primer | Purpose | Primer Sequence (5'→3') |
| --- | --- | --- |
| AaABF3-F | Clone | 5'-ATGAGTTCATTAATGAATCCCAAG-3' |
| AaABF3-R | Clone | 5'-CTACCAAGGTCCTGATAACGTTCTT-3' |
| AaABF3-QF679 | RT-Q-PCR | 5'-GCTGCTTTCAACTTCAACACTTC-3' |
| AaABF3-QR808 | RT-Q-PCR | 5'-ATAAATCACGTTGAGCCATGCT-3' |
| pAaABF3-F | Clone | 5'-TTCTCACCAGATCCCTTCCCTAG-3' |
| pAaABF3-R | Clone | 5'-CAAAGTCCTTCCCACTTCCACCC-3' |
| 1391Z-pAaABF3-PstI-F | GUS | 5'-TTACGCCAAGCTTGGCTGCAGAGTATAACTTGATATCGACGGTGT-3' |
| 1391Z-pAaABF3-BamHI-R | GUS | 5'-CCAGTGAATTCCCGGGGATCCTGCTTAATTCATCCAAACCTATC-3' |
| pHB-AaABF3-YFP-BamHI-F | Subcellular localization | 5'-CTCTCTCTCAAGCTTGGATCCATGAGTTCATTAATGAATCCCAAG-3' |
| pHB-AaABF3-YFP-SpeI-R | Subcellular localization | 5'-GCCCTTGCTCACCATACTAGTCCAAGGTCCTGATAACGTTCTT-3' |
| pB42AD-AaABF3-EcoRI-F | Y1H | 5'-GATTATGCCTCTCCCGAATTCATGAGTTCATTAATGAATCCCAAG-3' |
| pB42AD-AaABF3-XhoI-R | Y1H | 5'-AGAAGTCCAAAGCTTCTCGAGCTACCAAGGTCCTGATAACGTTCTT-3' |
| pALDH1-Box1-F | Y1H | 5'-AATTCGCACGCCACGTATGTATGCACGCCACGTATGTATGCACGCCACGTATGTATC-3' |
| pALDH1-Box1-R | Y1H | 5'-TCGAGATACATACGTGGCGTGCATACATACGTGGCGTGCATACATACGTGGCGTGCG-3' |
| pALDH1-Box2-F | Y1H | 5'-AATTCTTCTATCACGTAACCAATTCTATCACGTAACCAATTCTATCACGTAACCAAC-3' |
| pALDH1-Box2-R | Y1H | 5'-TCGAGTTGGTTACGTGATAGAATTGGTTACGTGATAGAATTGGTTACGTGATAGAAG-3' |
| pCold-AaABF3-BamHI-F | Prokaryotic expression | 5'-TCGGTACCCTCGAGGGATCCATGAGTTCATTAATGAATCCCAAG-3' |
| pCold-AaABF3-HindIII-R | Prokaryotic expression | 5'-GACTGCAGGTCGACAAGCTTCTACCAAGGTCCTGATAACGTTCTT-3' |
| ABF3-pALDH1-F | EMSA | 5'-GCCCAATACTCCAAAAGAGCACGCCACGTATGTATTGTAGATTTGTAGCC-3' |
| ABF3-pALDH1-R | EMSA | 5'-GGCTACAAATCTACAATACATACGTGGCGTGCTCTTTTGGAGTATTGGGC-3' |
| ABF3-pALDH1-mF | EMSA | 5'-GCCCAATACTCCAAAAGAGCACGCACGATATGTATTGTAGATTTGTAGCC-3' |
| ABF3-pALDH1-mR | EMSA | 5'-GGCTACAAATCTACAATACATATCGTGCGTGCTCTTTTGGAGTATTGGGC-3' |
| AaABF3-Fi | RNAi transgenic plants | 5'-CACCGGTGGTCATTGTCAGAATGGAGT-3' |
| AaABF3-Ri | RNAi transgenic plants | 5'-ATTCTGAAGCCTTCCATTTGTTC-3' |
| AaABF3-SacI-F | Overexpression transgenic plants | 5'-CGAGCTCATGAGTTCATTAATGAATCCCAAG-3' |
| AaABF3-XbaI-R | Overexpression transgenic plants | 5'-TGCTCTAGACTACCAAGGTCCTGATAACGTTCTT-3' |
| Actin-QF | RT-Q-PCR | 5'-CCAGGCTGTTCAGTCTCTGTAT-3' |
| Actin-QR | RT-Q-PCR | 5'-CGCTCGGTAAGGATCTTCATCA-3' |
| ADS-QF | RT-Q-PCR | 5'-AATGGGCAAATGAGGGACAC-3' |
| ADS-QR | RT-Q-PCR | 5'-TTTCAAGGCTCGATGAACTATG-3' |
| CYP71AV1-QF | RT-Q-PCR | 5'-CGAGACTTTAACTGGTGAGATTGT-3' |
| CYP71AV1-QR | RT-Q-PCR | 5'-CGAAGCGACTGAAATGACTTTACT-3' |
| DBR2-QF | RT-Q-PCR | 5'-GCGGTGGTTACACTAGAGAACTT-3' |
| DBR2-QR | RT-Q-PCR | 5'-ATAATCAAAACTAGAGGAGTGACCC-3' |
| ALDH1-QF | RT-Q-PCR | 5'-CAGTTTCTGACCCAAATCCAGGTTGA-3' |
| ALDH1-QR | RT-Q-PCR | 5'-TCGGAGTAGTTGGTCACAT-3' |
